# Numerical Simulation of Flow-Induced Noise in High Pressure Reducing Valve

**DOI:** 10.1371/journal.pone.0129050

**Published:** 2015-06-10

**Authors:** Lin Wei, Guorong Zhu, Jinyuan Qian, Yang Fei, Zhijiang Jin

**Affiliations:** 1 Institute of Process Equipment, Zhejiang University, Hangzhou, China; 2 Technical Development Department, Ningbo Special Equipment Inspection Center, Ningbo, China; Shanxi University, CHINA

## Abstract

The main objective of this paper is to study the characteristics of flow-induced noise in high pressure reducing valve (HPRV) and to provide some guidance for noise control. Based on computational fluid dynamics (CFD), numerical method was used to compute flow field. Ffowcs Williams and Hawkings Model was applied to obtain acoustic signals. The unsteady flow field shows that noise sources are located at the bottom of plug for valve without perforated plate, and noise sources are behind the plate for valve with perforated plate. Noise directivity analysis and spectrum characteristics indicate that the perforated plate could help to reduce noise effectively. Inlet pressure has great effects on sound pressure level (SPL). The higher inlet pressure will lead to larger SPL at high frequency. When the maximum Ma is close to 1, SPL at low frequency becomes very high.

## Introduction

High pressure reducing valves (HPRV) are widely used in industrial process. By regulating the steam pressure, HPRV helps to ensure normal operation of steam systems. Owing to the high speed of superheated steam and complex flow region, the noise and vibration in HPRV are severer than general control valve, but the relationship between noise and flow has not been fully understood. Hence, characteristics of the flow-induced noise in HPRV should be researched in preparation for better noise control.

Experimental methods are used to measure sound pressure level (SPL) and sound intensity. Nakano et al. [1] observed four typical flow patterns in a pressure reducing valve. It is shown that the jet flow along plug separates from the wall to form an annular jet impinging on the inner wall of the valve chest, and such flow oscillation causes the resonance in the chest cavity. Janzen et al. [2] tested the noise produced by a gate valve. The noise is caused by vortex shedding over the interior cavity in the valve, and the angle of chamfer next to the cavity has much influence on acoustic response. With piezoelectric pressure transducers, Li et al. [3] researched self-excited high frequency oscillations. Their results indicate that magnetic fluid can be used to overcome the self-excited oscillations and noises.

Under severe conditions, experimental method has difficulty in implementation and costs a lot. As the rapid development of computers and computational fluid dynamics (CFD) technologies, numerical method becomes a convenient method to obtain flow and acoustic characteristics. Ueno et al. [4] numerically researched the valve noise due to cavitation in incompressible laminar flow. Lafon et al. [5,6] used both pure Euler method and a boundary layer introduced method to calculate the noise in a shallow cavity. The results of the latter method are more close to experimental data. Ryu et al. [7] used a frequency domain acoustic analogy to compute the aerodynamic noise in a quick-opening throttle valve. It is found that anti-vortex lines form behind the valve during the quick-opening behavior, promote large scale turbulence, and generate dipolar sound. Wang et al. [8] put forward a numerical method based on Reynolds average Navier-Stokes (RANS) equation and *k-ε* equation to investigate acoustic distribution due to flow of inner leakage in ball valve. The turbulent flow was proved to be the main cause of jet noise. Sun et al. [9–13] researched the effects of noise on the pattern formation in an epidemic model. Tamura et al. [14] proposed a new function which consists of distribution function and equilibrium distribution function to improve the computational efficiency of finite difference lattice Boltzmann method (FDLBM). The flow acoustic resonance at safety relief valve (SRV) stub pipes was simulated and it is found that the noise was also generated by unsteady vortices. Most studies are about incompressible flow-induced noise, however, noise induced by compressible high velocity flow in valve has not been fully explored.

At present there are two ways to reduce the noise in HPRV, that is, the source treatment and the path treatment [15]. Source treatment can be realized by arranging a trim, perforated plate or other special elements in a control valve. Smith and Luloff [16] proposed that the noise could be eliminated by chamfering valve seats. Youn et al. [17] analyzed the flow in radial slit structure with CFD 2000 software and Schlieren photography method. The experimental results indicated that the radial structure can reduce noise level by 40 dB. Different kinds of perforated plates are used to reduce noise in valves, but the relevant mechanism researches are not sufficient.

In the present paper, numerical simulation was carried out to obtain complex compressible flow field of high temperature and pressure steam, and to analyze the flow-induced noise in HPRV. The effects of perforated plate on noise control in HPRV were analyzed by comparing performances of valve with a perforated plate and valve without. Finally, different inlet pressure was applied to discuss the effects of pressure on noise at different frequency.

## Computational Model

### Geometrical structure

**Fig 1:**
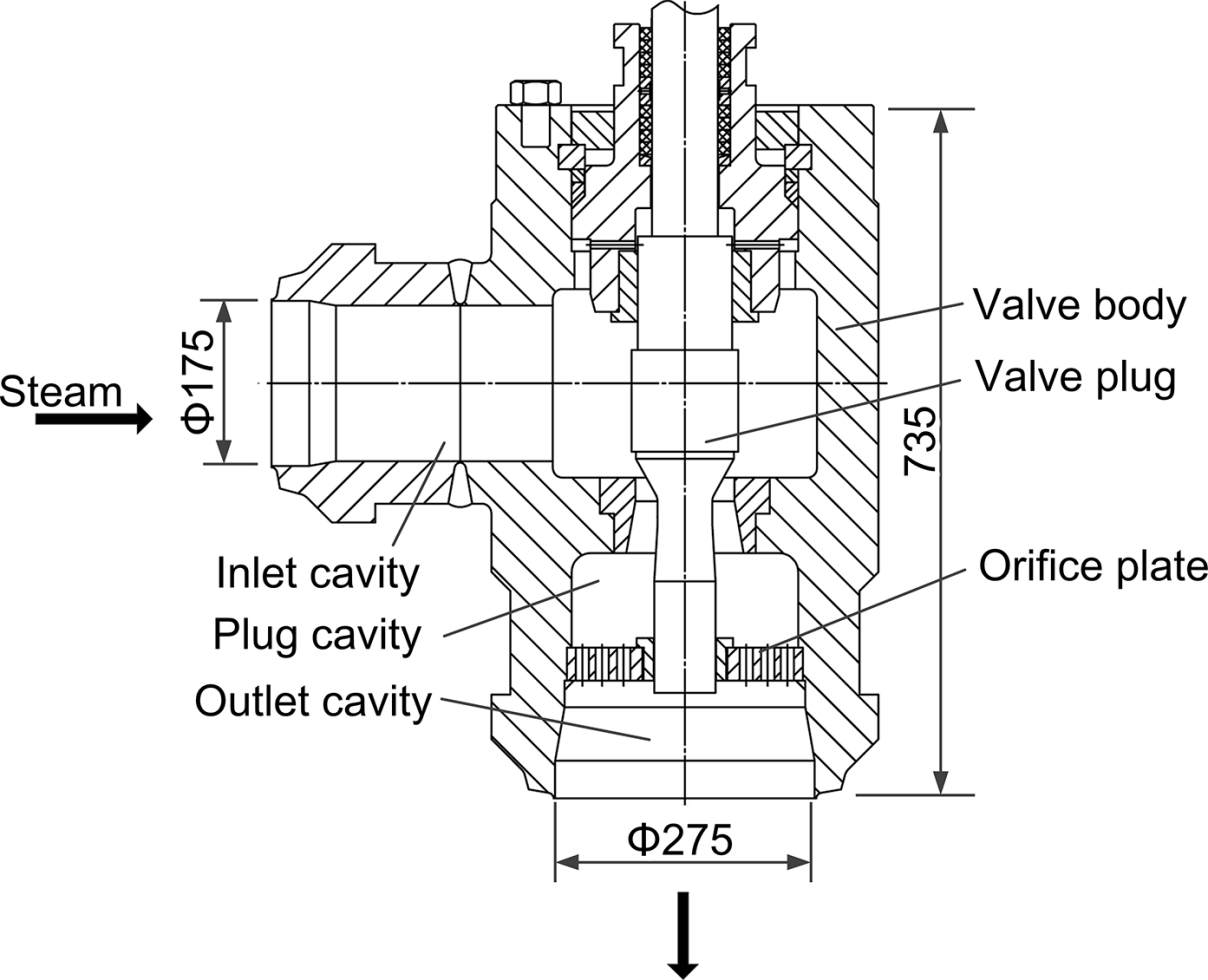
Structure diagram of HPRV (Valve B). The HPRV mainly consists of an angle-type valve body, a conical plug and a perforated plate. The flow region includes three cavities: inlet cavity, plug cavity and outlet cavity.

The HPRV in Fig 1 [18] mainly consists of a forge-welded angle-type valve body, a conical plug and a perforated plate used for noise control. The perforated plate is a rounded flat plate with many through holes. By adjusting displacement of valve plug, HPRV keeps the outlet pressure at target value. The flow region includes three cavities: inlet cavity, plug cavity and outlet cavity. Fluids from inlet flow to inlet cavity, and then through the valve plug turn 90° into plug cavity, finally get through perforated plate and outlet cavity. In order to observe the effects of perforated plate, a model without perforated plate was built while other structural parameters are same as Fig 1. HPRV without perforated plate and HPRV with perforated plate are labeled as Valve A and Valve B, respectively. The diameter of inlet and outlet are Φ175 mm and Φ275 mm.

### Grid and boundary conditions

**Fig 2:**
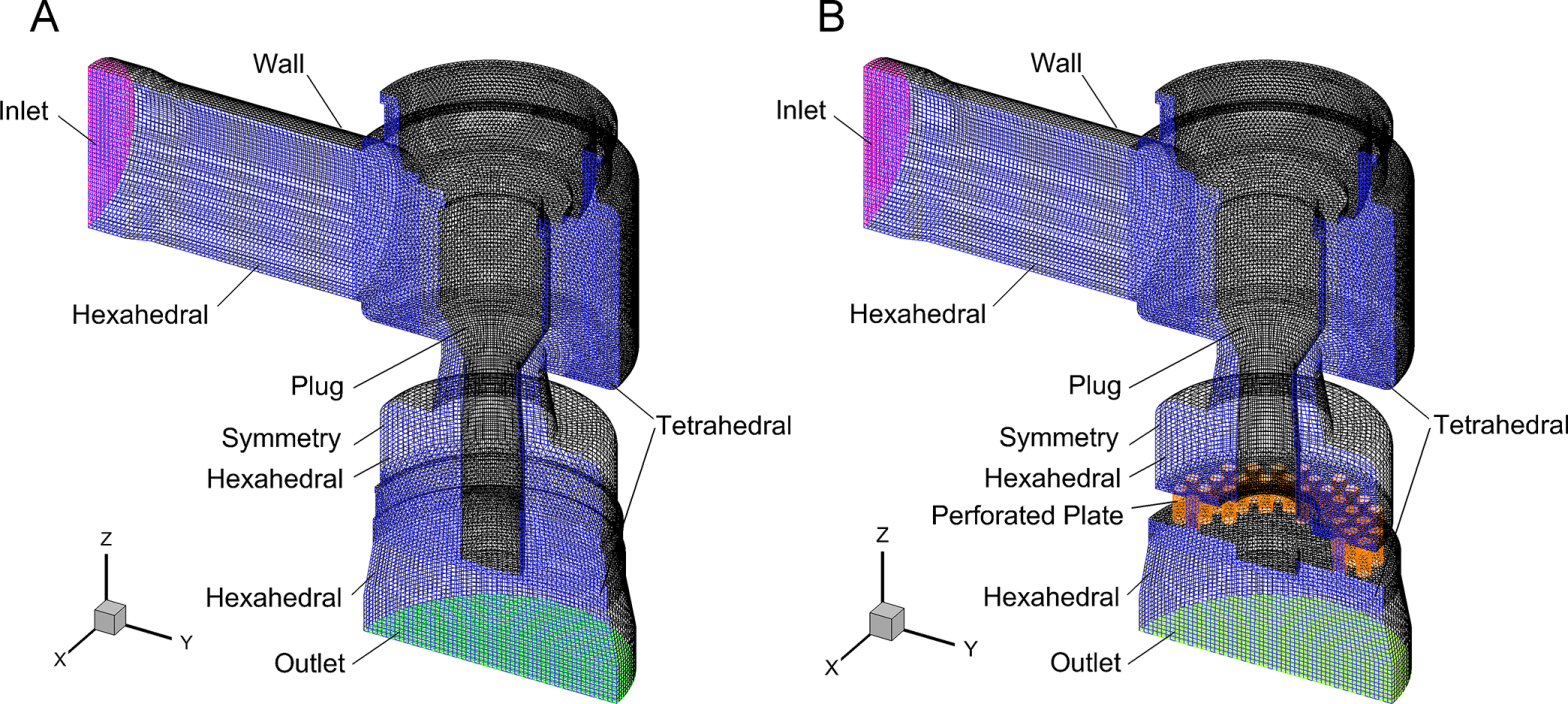
Grids of flow zone in HPRVs: (a) Valve A; (b) Valve B. The flow zone was meshed with hexahedral and tetrahedral cells. Pressure of inlet and outlet were given and surface *x* = 0 was set as symmetry. Other surfaces were set as wall.

Three-dimension models were built for HPRVs of 60% opening. To improve the computational efficiency, half of the symmetrical structure was employed. The pre-processing software Gambit was used to generate grids. The computational region was split into six connected parts. Four parts were meshed with structured hexahedral cells and the other two parts were meshed with unstructured tetrahedral cells as shown in Fig 2.

**Fig 3:**
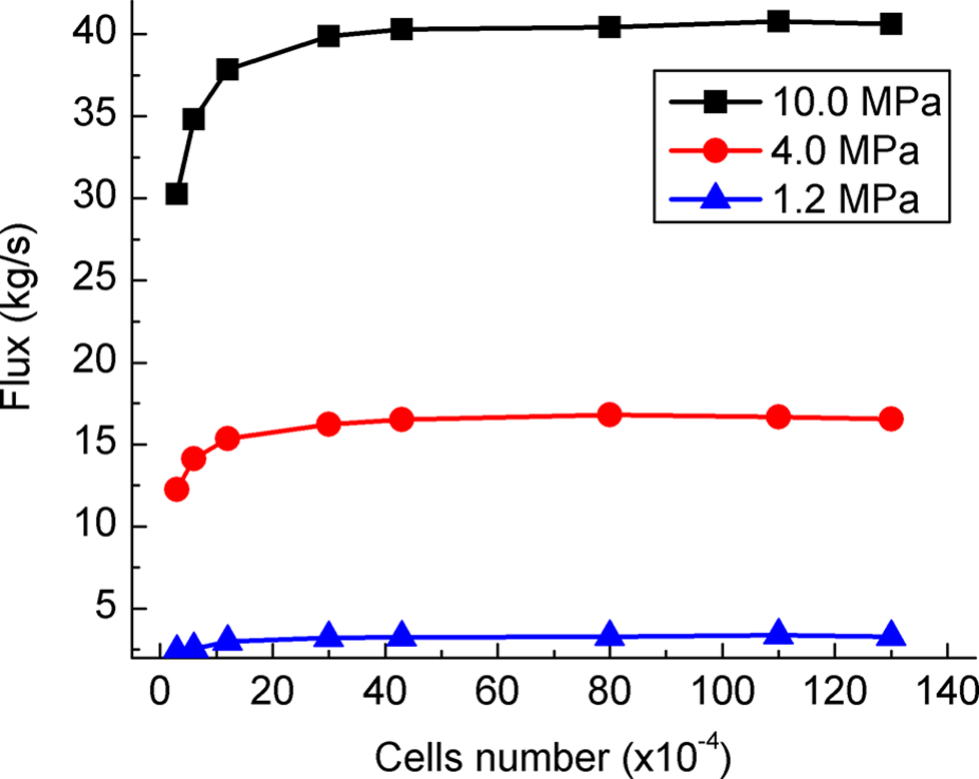
Grid independence check. For different inlet pressure, flux has same trends with cells number.

The grid independence check of Valve B is listed in Table 1 [18].Those grids were meshed with cells of different size. Fig 3 shows flux variation with cells number for different inlet pressure conditions. It indicates that the mass flux difference is less than 4% for cells number > 300 000. Grid 5 of about 430 000 cells was used considering both accuracy and computation time. The model of Valve A was meshed with cells of same size as Valve B.

**Table 1:** Grid independence check.

Grid	Cell size (mm)	Cells number
1	12	30 000
2	10	60 000
3	8	120 000
4	6	300 000
5	4.5	430 000
6	3.6	800 000
7	3.2	1100 000
8	3	1300 000

The pressure of inlet and outlet are set to solve the compressible flow and specific boundary conditions of the computational domain are:

1. Inlet: pressure inlet of *p*_in_ = 1x10^7^ Pa and *T* = 813 K.
2. Outlet: pressure outlet of *p*_out_ = 1x10^6^ Pa and *T* = 813 K.
3. Surface *x* = 0 is set as symmetry.
4. Other surfaces are set as wall and no-slip condition is used.

## Numerical Method

### Governing equations of flow

Turbulent model plays an important role in the simulation of gas flow and noise distribution. For the prediction of flow pattern inside bubble column, Large Eddy Simulation (LES) is more accurate for prediction of gas hold-up, velocity and swirling flow than standard *k*–*ε*model, Reynolds Stress Model (RSM) [19, 20]. And Silva et al. [21] pointed out that *k*–*ε* turbulence model reached good results for gas holdup and velocity profiles in fully developed region, while RSM gets a good flow field for the gas holdup mainly in the sparger region.

The fluctuations of sound pressure are much smaller than that of fluid pressure in simulation of noises. However, LES model is able to capture broadband noise in simulation of flow. The LES equations of motion describe the evolution of large scales with the effect of the smaller unresolved scales being modeled, thereby, providing time-dependent quantification of the noise sources [22]. There have been a lot of achievements in predicting aero-acoustic characteristics by directly coupling LES predictions on an interfacing surface with the Kirchoff or the Ffowcs Williams and Hawkings (FW-H) methods [23].

In HPRV, the compressibility of high temperature and pressure steam cannot be ignored. And LES method is used to solve the compressible filtered Navier-Stokes equations for ideal gas. A filtered variable (denoted by an overline) is defined by

$$\bar{f}(x,t)=\int_{\Omega }f(x\prime ,t)G(x-x\prime )\text{d}x\prime ,$$ (1)

where **x** = (*x*, *y*, *z*) are the coordinates of the cell center; *t* is the time; *Ω* is the fluid domain; and *G* is the filter function that determines the scale of the resolved eddies. In FLUENT the finite-volume discretization implicitly provides the filtering operation:

$$\bar{f}(x,t)=\frac{1}{V}\int_{V}f(x\prime ,t)\text{d}x\prime ,\;x\prime \in V,$$ (2)

where *V* is the volume of a computational cell. The filter function *G* implied here is

G(x,x′)={1/V,x′∈V0,x′otherwise. (3)

The conservative form of the continuity, momentum and energy equations can be expressed as

$$\frac{\partial \bar{Q}}{\partial t}+\frac{\partial {\bar{F}}_{i}^{inv}}{\partial x_{i}}-\frac{\partial {\bar{F}}_{i}^{vis}}{\partial x_{i}}=0.$$ (4)

The conservative variables are defined as $\bar{Q}={\left[\bar{\rho },\bar{\rho }{\bar{u}}_{1},\bar{\rho }{\bar{u}}_{2},\bar{\rho }{\bar{u}}_{3},\tilde{E}\right]}^{T}$, where *ρ* is the density; *u* is the velocity. The total energy is $\tilde{E}=\bar{\rho }\tilde{e}+\bar{\rho }{\tilde{u}}_{i}{\tilde{u}}_{i}/2$ and $\tilde{e}$ is the internal energy. The inviscid and viscous fluxes are respectively given by ${\bar{F}}_{i}^{inv}={\tilde{u}}_{i}\bar{Q}+{\left[0,\delta_{1i}\bar{p},\delta_{2i}\bar{p},\delta_{3i}\bar{p},\bar{p}{\tilde{u}}_{i}\right]}^{T}$ and ${\bar{F}}_{i}^{vis}={\left[0,{\tilde{\tau }}_{1i},{\tilde{\tau }}_{2i},{\tilde{\tau }}_{3i},{\tilde{\tau }}_{ki}{\tilde{u}}_{k}+{\tilde{q}}_{ii}\right]}^{T}$, where *δ*_*ij*_ is the Kronecker delta; $\bar{p}$ is the static pressure. The stress tensor ${\tilde{\tau }}_{ij}$ and the heat flux ${\tilde{q}}_{i}$ are formulated as

$${\tilde{\tau }}_{ij}=2(\mu +\mu_{T})({\tilde{S}}_{ij}-\frac{1}{3}\frac{\partial {\tilde{u}}_{j}}{\partial x_{j}}\delta_{ij}),$$ (5)

$${\tilde{q}}_{i}=-(\kappa +\kappa_{T})\frac{\partial \tilde{T}}{\partial x_{i}},$$ (6)

where *μ* and *μ*_*T*_ are the molecular viscosity and the eddy viscosity; ${\tilde{S}}_{ij}$ is the strain rate tensor; *κ* and *κ*_*T*_ are the thermal diffusivity and the eddy thermal diffusivity.

For ideal gas the relation between pressure, density and temperature is defined as the state equation:

$$\bar{p}=\bar{\rho }R\tilde{T}.$$ (7)

### Ffowcs Williams and Hawkings Model

FW-H Model based on Lighthill’s acoustic analogy was used to compute acoustic signal. The FW-H equation is an inhomogeneous wave equation that can be derived by manipulating continuity equation and Navier-Stokes equations:

$$\frac{1}{a_{0}^{2}}\frac{\partial^{2}p\prime }{\partial t^{2}}-\nabla^{2}p\prime =\frac{\partial^{2}}{\partial x_{i}\partial x_{j}}\left\{T_{ij}H\left(f\right)\right\}-\frac{\partial }{\partial x_{i}}\left[P_{ij}n_{j}+\rho u_{i}u_{n}\delta \left(f\right)\right]+\frac{\partial }{\partial t}\left[\rho u_{n}\delta \left(f\right)\right],$$ (8)

where *u*_*i*_ is the fluid velocity component in *x*_*i*_ direction; *u*_*n*_ is the fluid velocity component normal to the source surface *f* = 0; *δ* (*f*) is the Dirac delta function; *H* (*f*) is the Heaviside function; *p*′ is the sound pressure at the far field (*p*′ = *p-p*_0_); *a*_0_ is the far field sound speed; *T*_*ij*_ is the Lighthill stress tensor; and *P*_*ij*_ is the compressive stress tensor.

**Fig 4:**
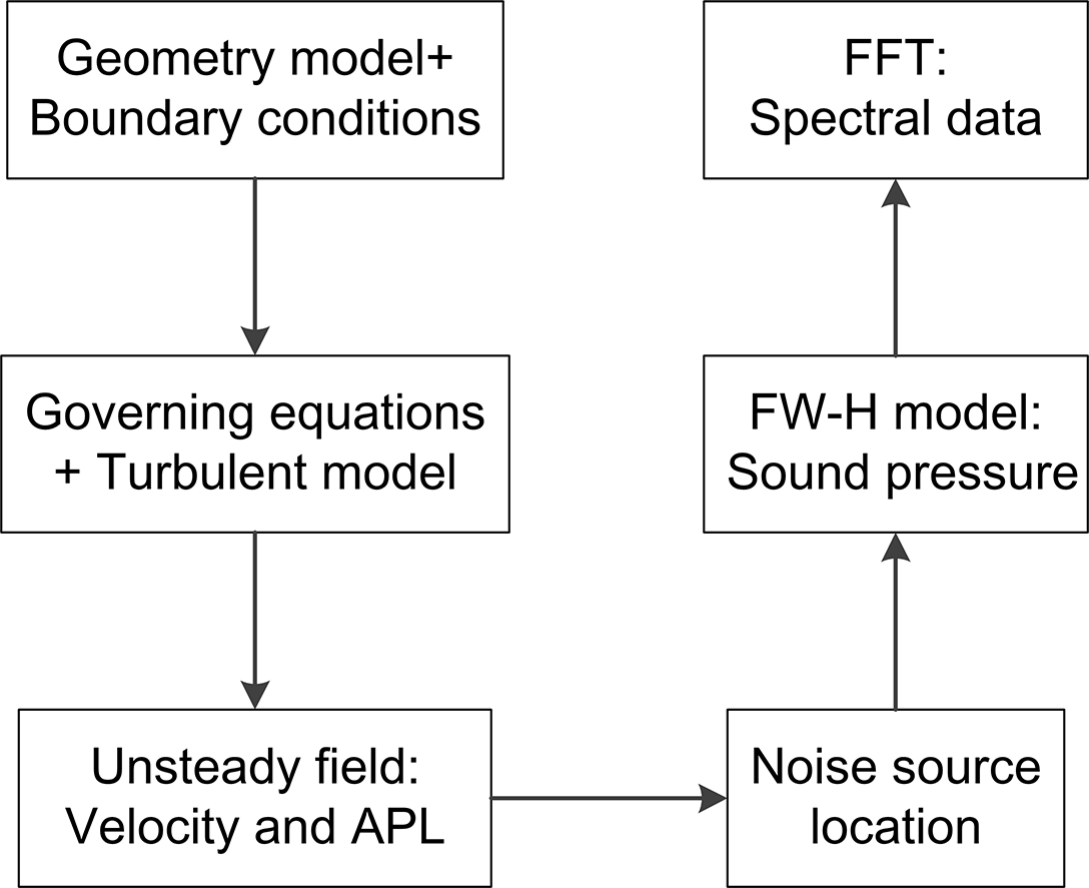
Flow chart of simulation.

The flow chart of CFD simulation is shown in Fig 4. Based on governing equations and corresponding boundary conditions, the unsteady flow field was firstly obtained. According to distribution of acoustic power level (APL), the noise sources can be located. Then the FW-H model was activated and unsteady flow was calculated. By monitoring sound pressure signals at receivers around the noise sources and Fast Fourier Transform (FFT), spectral data were achieved for analysis of noise directivity and spectral characteristics.

## Results and Discussion

### Unsteady flow

**Fig 5:**
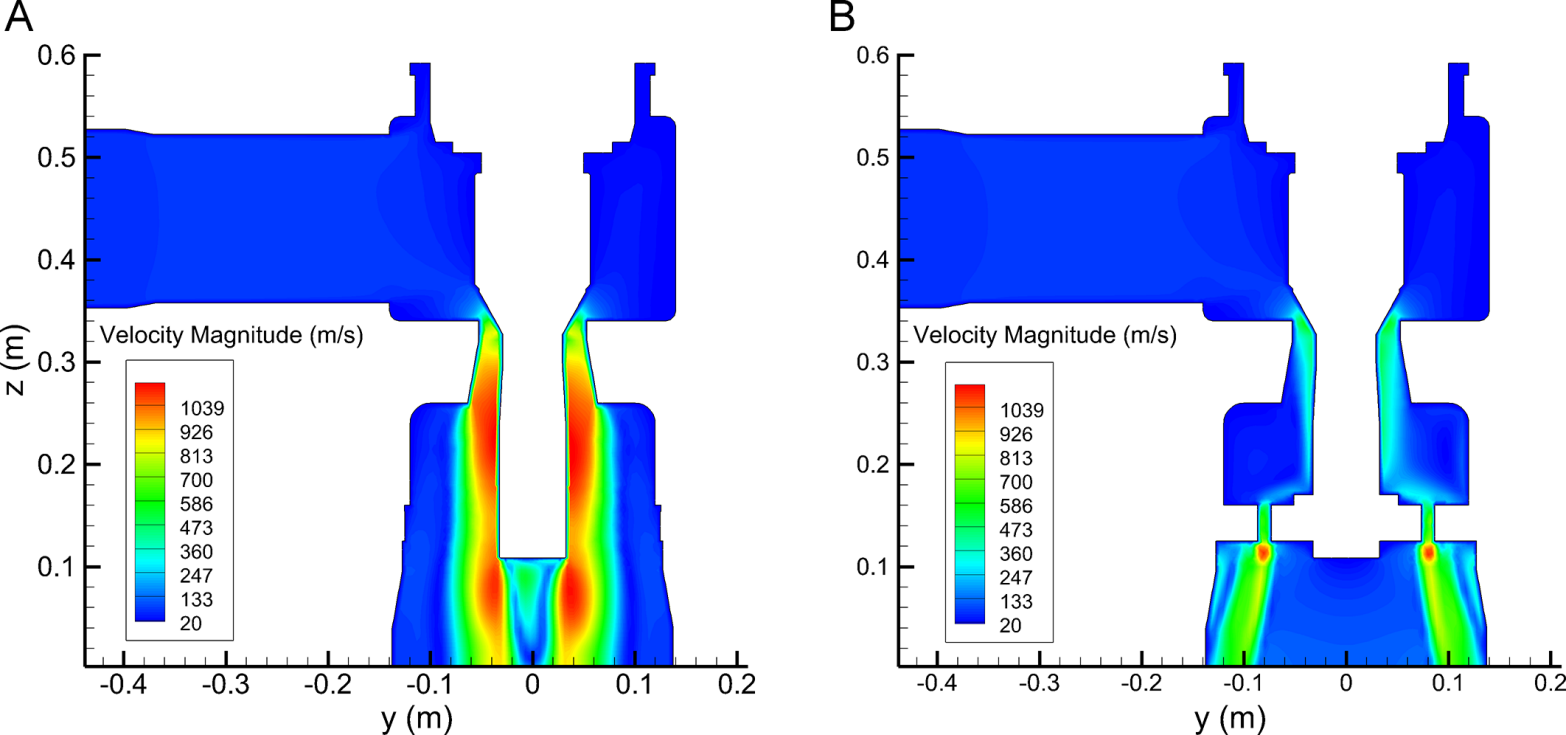
Velocity contour on plane *x* = 0 mm: (a) Valve A; (b) Valve B. High velocity occurs mainly around the valve plug and at the outlet cavity.

**Fig 6:**
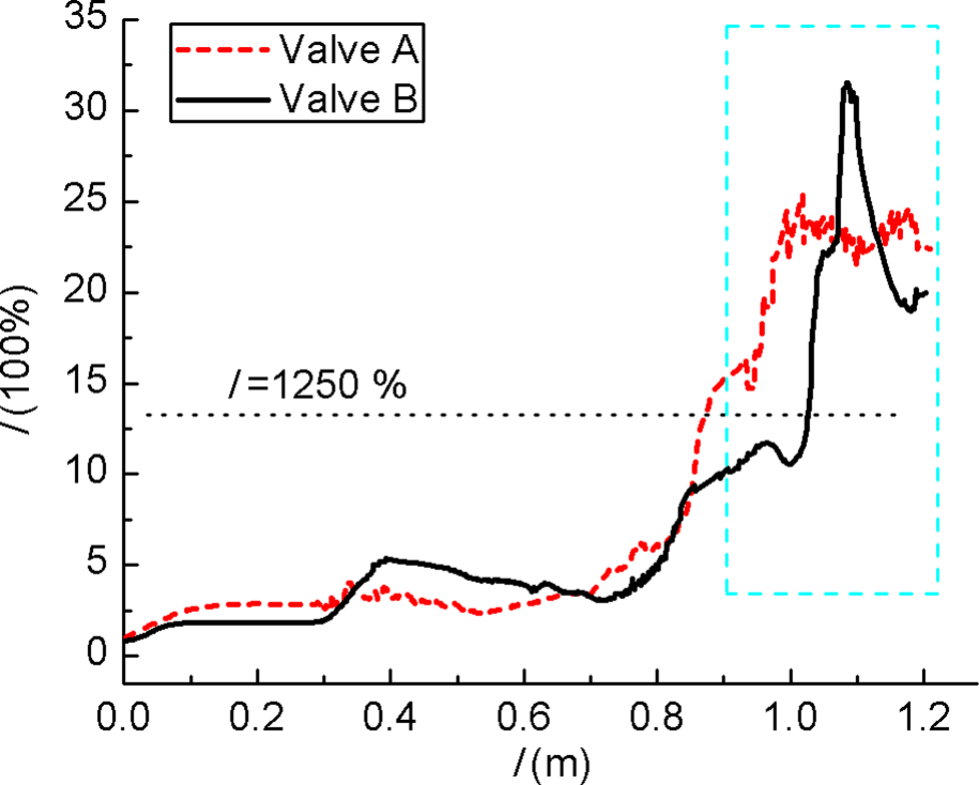
Turbulent intensity variation along streamlines. For *l* = 0.9~1.2 m (from perforated plate to outlet) turbulent intensity is much larger than other region.

**Fig 7:**
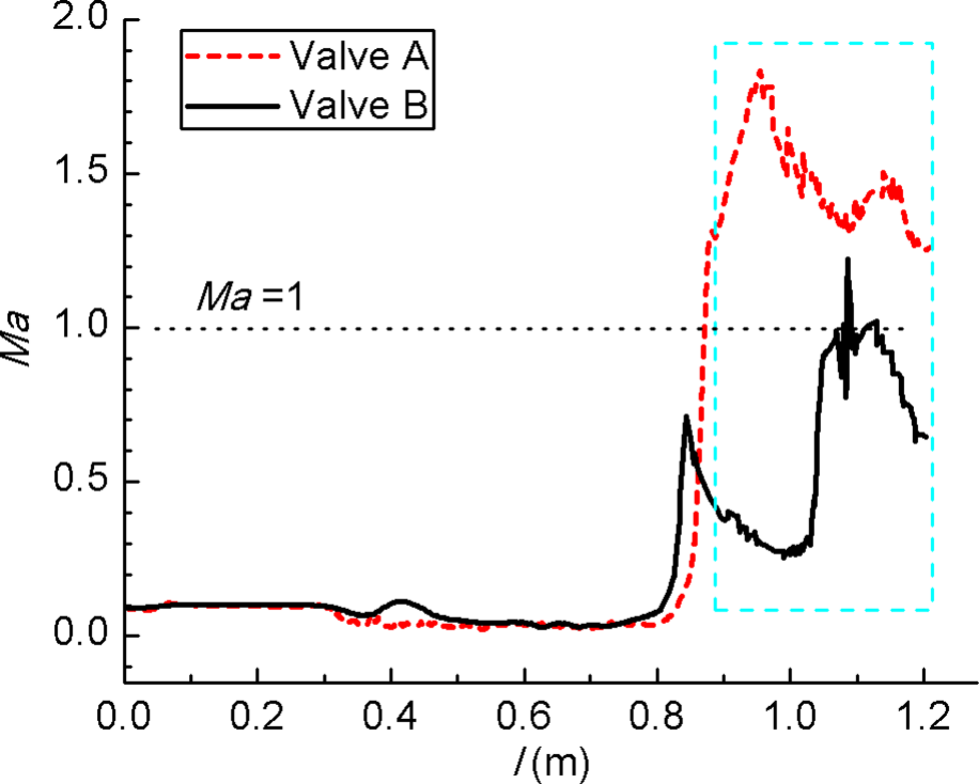
Mach number variation along streamlines. For *l* = 0.9~1.2 m, Ma is higher than other region and Ma > 1 indicates that the flow is supersonic.

Admittedly, the flow status has much influence on acoustic field. In Fig 5 high velocity occurs mainly around the valve plug and at the outlet cavity. It can be seen that the high velocity area of Valve A is wider than Valve B. Velocity at rest part is much lower. Fig 6 shows turbulent intensity variation along streamline for the two models. When streamline length *l* > 0.8 m, turbulent intensity increases fast. For *l* = 0.9~1.2 m (from perforated plate to outlet) turbulent intensity is much larger than other region where the turbulent intensity is kept under 1250%. This is because that steam expands in the outlet cavity and causes great disturbances. The difference of Mach number (Ma) between the two models is clearly in Fig 7. Ma at *l* = 0.9~1.2 m is higher than other region and Ma > 1 indicates the supersonic flow. Ma of Valve B is 30~60% lower than Valve A.

**Fig 8:**
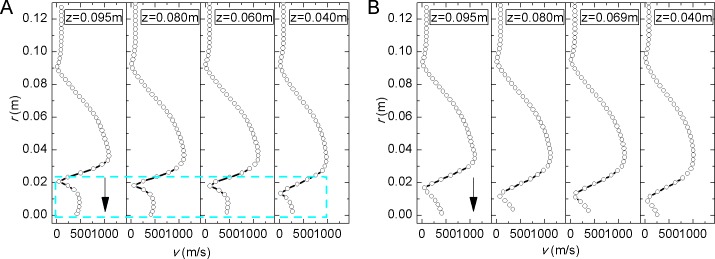
Velocity distribution of Valve A at (a) *φ* = -120° section and (b) *φ* = 120° section. *φ* is the angle from *x* axis. *φ* = -120° and *φ* = 120° are symmetric about *y* =0 plane. At different *z* there is a big velocity peak which becomes flat with *z* decreases.

The velocity distribution of Valve A on sections of different *z* is shown in Fig 8 where *φ* is the angle from *x* axis. *φ* = -120°and *φ* = 120° are symmetric about *y* = 0 plane. There is a big velocity peak in each graph. Although the outlet part is axisymmetric the velocity near the inlet side is higher, which is indicated by the small peak in dashed box of Fig 8A. As *z* decreases the small peak becomes flat and extends in the direction of the arrow. On both sections the big peak also becomes flat with *z* decreasing.

**Fig 9:**
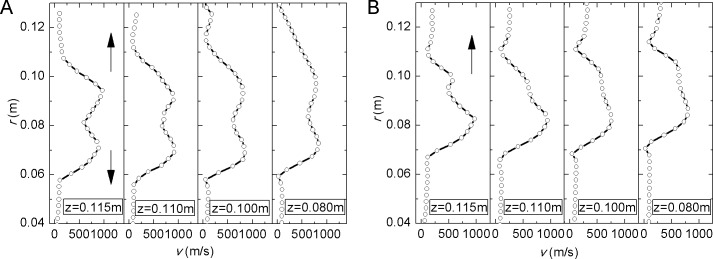
Velocity distribution of Valve B at (a) *φ* = 120° section and (b) *φ* = 150° section. Peaks in the velocity distribution are caused by jet flow through the holes. As *z* decreases the peaks become flat and extend in the direction of the arrows.

The velocity distribution of Valve B on different sections is shown in Fig 9. The curves are all behind the perforated plate. Because of the two jet flow through the holes there are two velocity peaks in Fig 9A. As *z* decreases the two peaks become flat and extend in the direction of the arrows. Since there is only one jet flow along radius at *φ* = 150° section, in Fig 9B the small peak next to the big one is induced by jet from adjacent holes.

The peaks of velocity curve indicate high velocity behind the holes because the sudden reduction of flow area in holes. Regions of the high velocity of each hole expand radially and even cross. The high velocity flow and expansion could cause complex turbulence and noise.

### Location of noise sources

**Fig 10:**
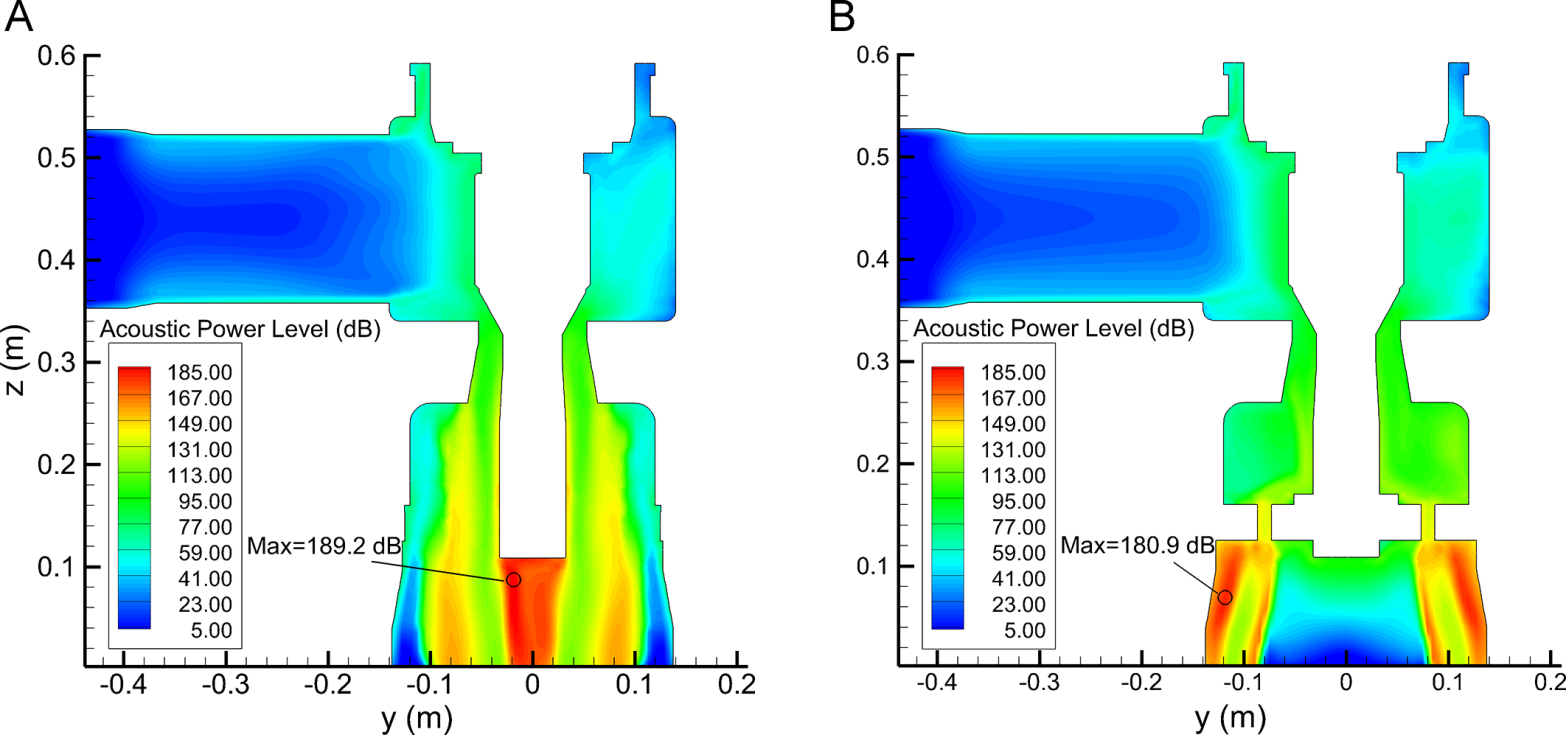
APL contour on plane *x* = 0 mm: (a) Valve A; (b) Valve B. For Valve A (A), at bottom of valve plug the maximum APL is 189.2 dB. For Valve B (B), behind the plate and near the wall, the maximum APL is 180.9 dB.

Fig 10 shows APL distribution on plane *x* = 0 mm. It can be seen that APL is below 50 dB in the inlet cavity, and when steam flows through valve plug, the noise increases rapidly. In Valve A (Fig 10A) APL at bottom of valve plug is very high and the maximum is 189.2 dB. In Valve B (Fig 10B) the steam velocity is reduced by perforated plate. So behind the plate and near the wall, the maximum APL is reduced to 180.9 dB.

**Fig 11:**
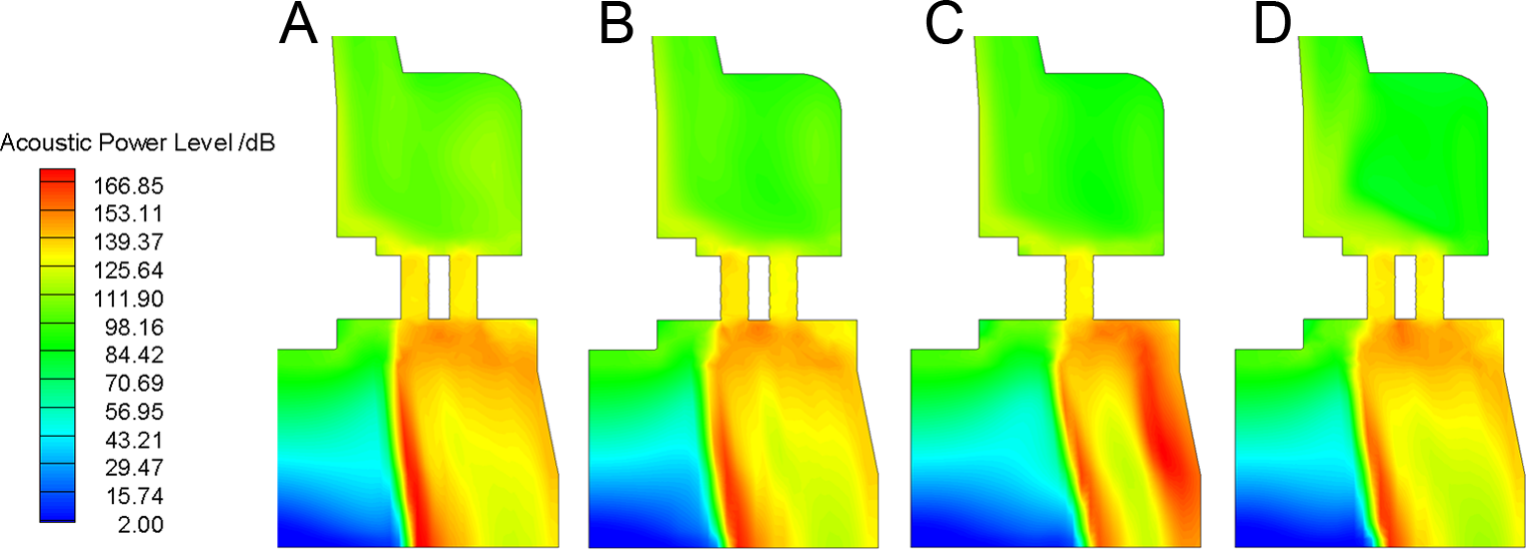
APL contour of different angles: (a) *φ* = 120°; (b) *φ* = 180°; (c) *φ* = -150°; (d) *φ* = -120°. APL of sections with two holes (A, B and D) is smaller than APL of section with one hole (C) since the flow is uniform in the radial direction for the former.

APL distribution of Valve B at different angle is shown in Fig 11. The highest noise occurs at the outlet cavity where turbulent vortexes continually shed from jet flow through the plate. APL is low in the center of outlet cavity because of backflow. Near the valve body wall, APL of sections with two holes is smaller than APL of section with one hole since the flow is uniform in the radial direction. The unsteady results indicate that the noise sources mainly locate at the bottom of the plug for Valve A and behind the plate for Valve B.

### Spectral characteristics

**Fig 12:**
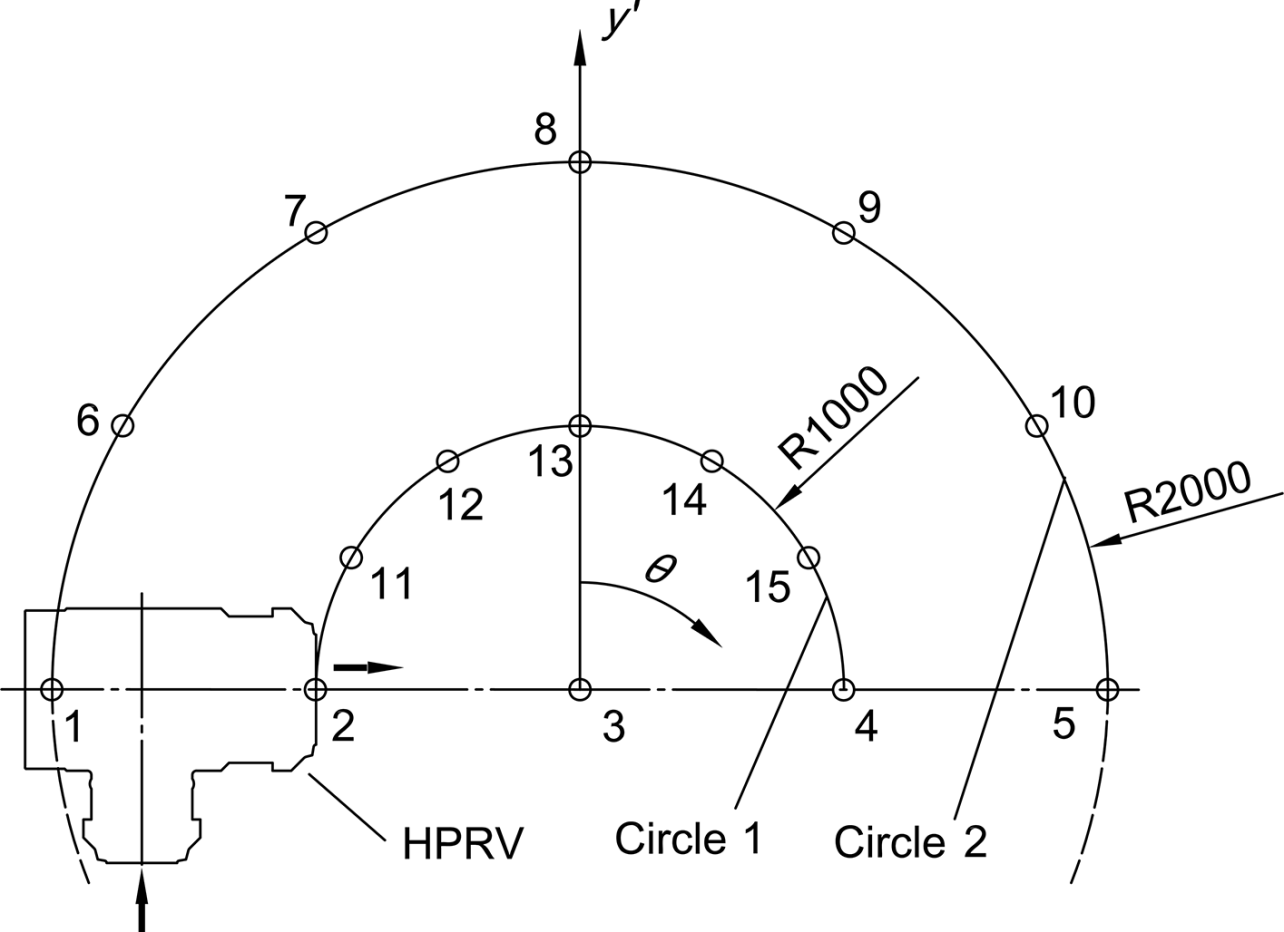
Arrangement of receiver points.

To analyze the noise directivity, the receiver points were arranged near the noise sources as shown in Fig 12. Around point 3 there are two circles, of which the radius are *R* = 1000 mm (Circle 1) and *R* = 2000 mm (Circle 2). On each circle twelve receiver points are distributed uniformly. Five receiver points are along the outlet direction every 1000 mm. By processing sound pressure signals at receivers with FFT, overall sound pressure level (OSPL) and frequency spectrums were obtained.

**Fig 13:**
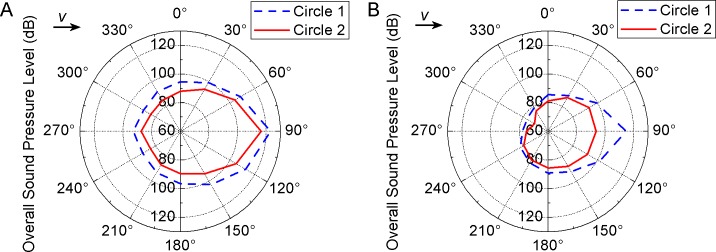
Noise directivity of (a) Valve A and (b) Valve B. The directivity curves have elliptical shapes and the downstream noise (*θ* = 90°) is larger than other directions.

Fig 13 shows the noise directivity expressed by OSPL of receiver points. The directivity curves have an elliptical shape. The downstream noise (*θ* = 90°) is larger than other directions. For Valve A (Fig 13A), Circle 2 has similar shape as Circle 1 but smaller values. In Fig 13B the directivity curve of Circle 2 is more similar to a circle since OSPL of *θ* = 90° is lower. Because of the angle type structure of valve body, OSPL of left side (*θ* = 90~270°) is higher than that of right side. This phenomenon is more clearly shown in Valve B.

**Fig 14:**
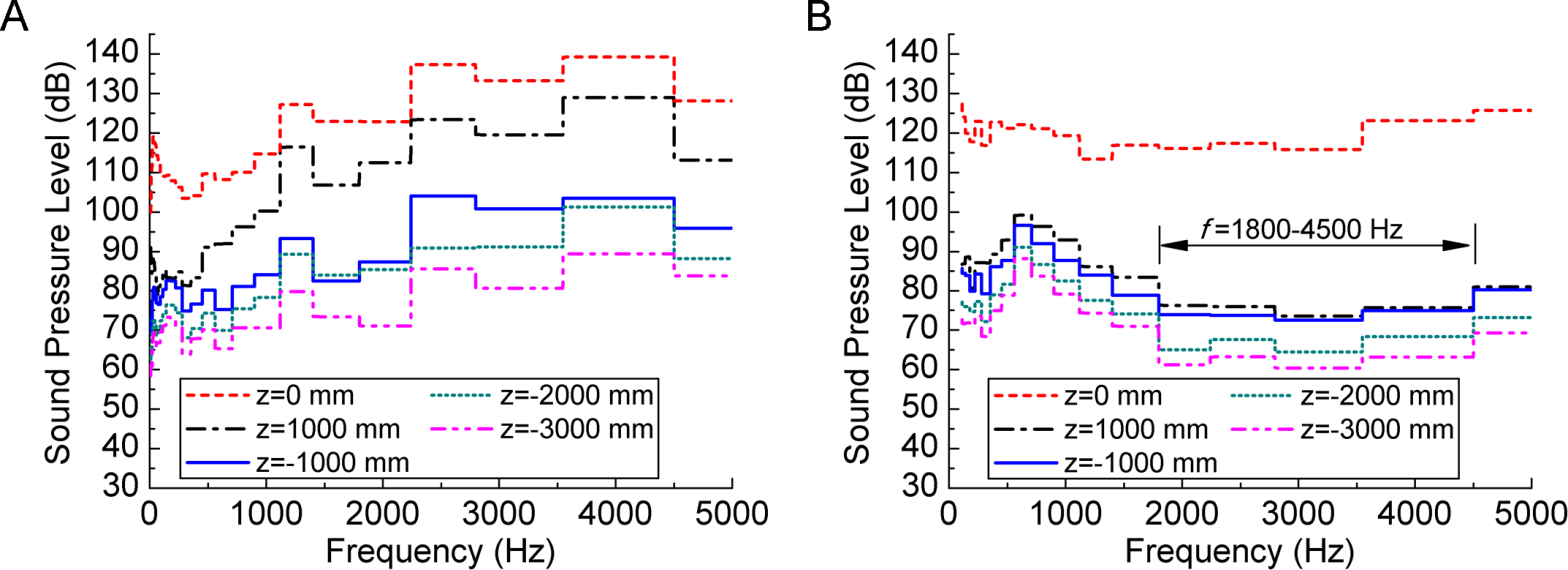
1/3-ocatave band curves of (a) Valve A and (b) Valve B. The flow-induced noise has a broadband property. Perforated plate of Valve A (A) is effective in reducing noise of *f* = 1800~4500 Hz.

1/3-octave band curves were obtained as shown in Fig 14. The flow-induced noise has a broadband property and curve of five points has same shape for each valve. Because outlet is at *z* = 0 mm plane, |*z*| of those five points is their distance from outlet. In Fig 14A SPL of each line increases gradually until 1000 Hz, and then it remains stable. Points of larger |*z*| have lower SPL curve. For Valve B in Fig 14B, SPL curve of *z* = 0 mm point (outlet) is at a higher level while other four curves are relatively low with narrow gap. Comparing the two graphs, it can be seen that the perforated plate is effective in reducing noise of *f* = 1800~4500 Hz. Table 2 shows OSPL of the five points along the outlet and *η* = OSPL_A_/OSPL_B_-1. OSPL_B_ is 1~30% lower than OSPL_A_ at different points. In Valve B, 1000 mm away from the outlet, OSPL attenuates fast with a speed of 30%. And the attenuation speed is 3% from 1000 mm to 2000 mm and 5% from 2000mm to 3000 mm.

**Table 2:** Overall sound pressure level.

Point	*z* (mm)	OSPL_A_ (dB)	OSPL_B_ (dB)	*η*
1	-1000	130.72	100.43	30.16%
2	0	142.53	140.34	1.56%
3	1000	108.31	99.23	9.15%
4	2000	102.66	96.64	6.23%
5	3000	92.74	91.85	0.97%

**Fig 15:**
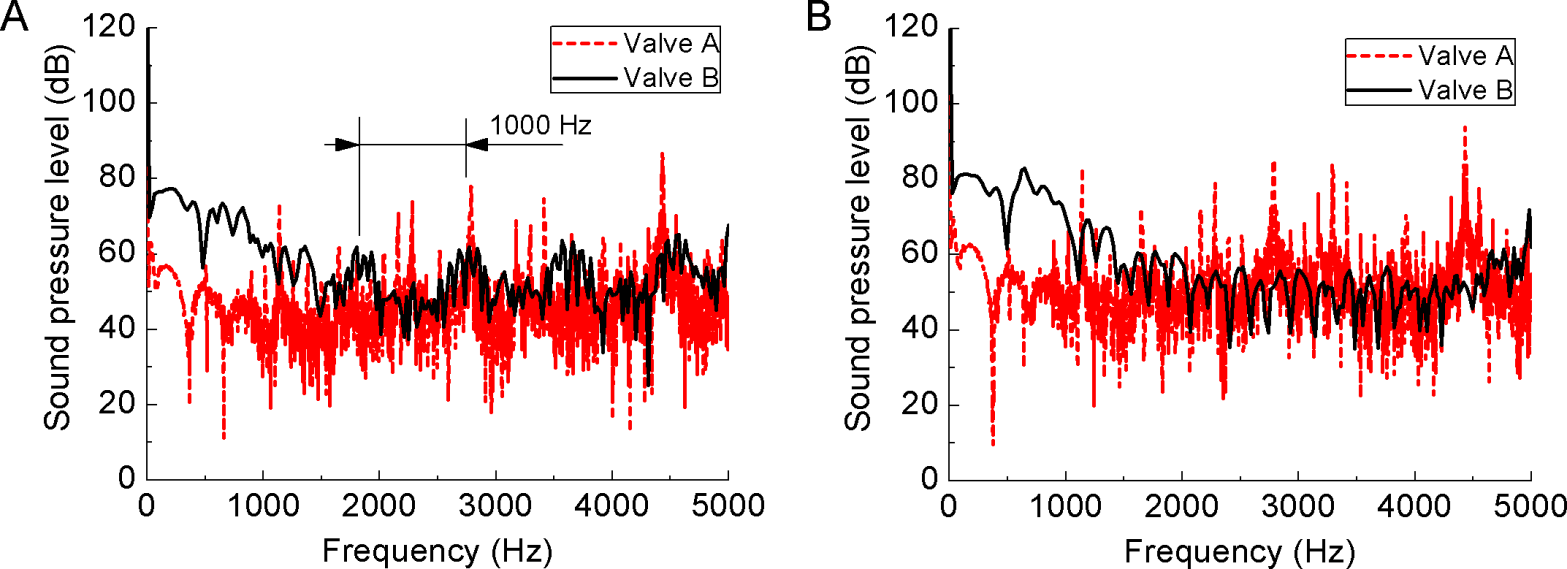
Frequency spectrums at (a) point 8 and (b) point 13. The acoustic signal of Valve A is random while the acoustic signal of Valve B is a periodic curve at point 8 (A). The spectral curves of Valve B is lower than Valve A at both points.

Fig 15 compares the frequency spectrums of both valves at points 8 and 13. The acoustic signal of Valve A is random while the acoustic signal of Valve B is a periodic curve (Fig 15A) consistent with the spectral property of reactive chamber mufflers. The spectral curves of Valve B is lower than Valve A at both points, because sound waves of some frequecy are reflected by the perforated plate.

### Effects of pressure

For general steam pipeline valves, steam pressure is less than 3 MPa [1, 2, 8, 11, 24, 25] and Ma is much lower than 1 [5]. However, in modern power plants, the steam inlet temperature and pressure through fully open valve can reach as high as 500°C and 20 MPa [26, 27]. To study the effects of pressure for various applications, different inlet pressure values of 1.2~10 MPa were applied.

**Fig 16:**
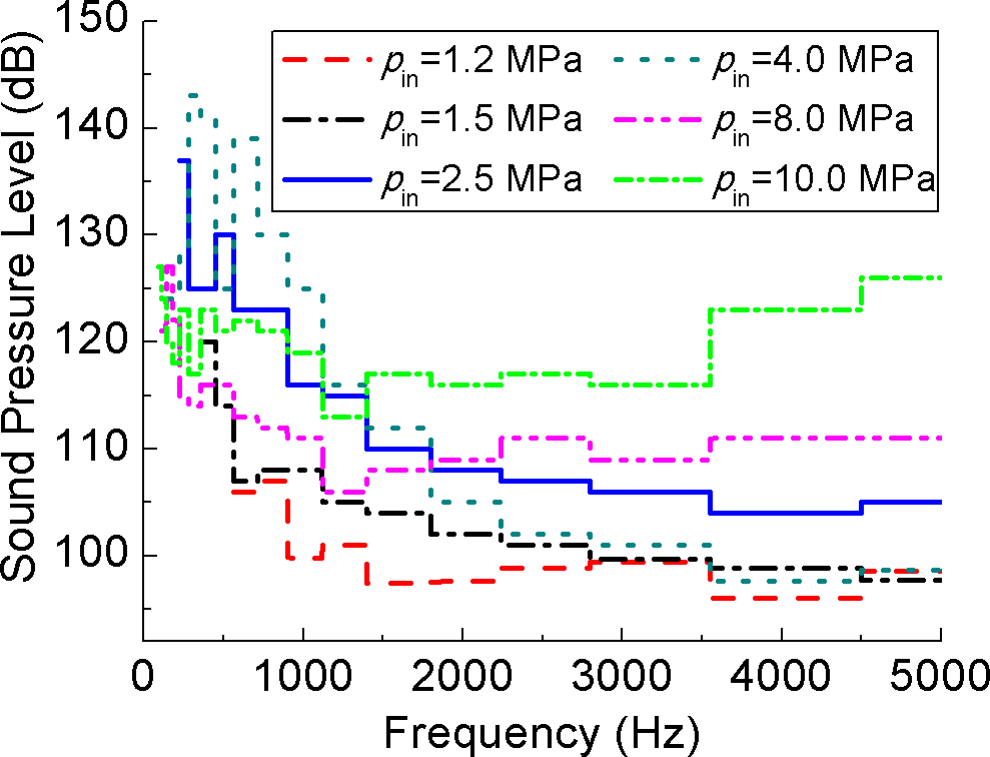
1/3-ocatave band curve of different inlet pressure. From 1500-5000 Hz the trend is obvious that the higher inlet pressure will cause the larger SPL. For *p*_in_ = 2.5 and 4.0 MPa, Ma is close to 1, and SPL is very high at low frequency.

Steam velocity is closely related to inlet pressure. For *p*_in_ = 2.5 MPa, the maximum Ma is 0.92. When *p*_in_ ≥ 4 MPa, the maximumMa is larger than 1 (equals to 1.16, 2.01, 2.17, respectively) and the flow turns into supersonic flow. The 1/3-ocatave band curves are shown in Fig 16. From 1500–5000 Hz the trend is obvious that the higher inlet pressure will cause the larger SPL. Curves of *p*_in_ = 1.2, 1.5, 2.5, 4 MPa are decreasing gradually with frequency, and SPL of low frequency is large. While for the other two curve, the SPL of high frequency is large as well as that of low frequency. In other words, the higher *p*_in_ leads to larger SPL of high frequency. For *p*_in_ = 2.5 and 4.0 MPa, Ma is more close to 1, and it shows that SPL is very high SPL at low frequency.

## Conclusions

Considering compressibility of steam, this work compared flow and noise property of two HPRVs with numerical simulation. The noise directivity, spectral characteristics of flow-induced noise were obtained and effects of pressure were analyzed.

The unsteady results show that noise sources of Valve A occurs at the bottom of the valve plug and noise sources of Valve B occurs behind the perforated plate. From the directivity analysis and the spectrum characteristics, the downstream noise of Valve B is smaller than Valve A especially at longer distance. The spectrum of Valve B is kind of periodic because of sound reflection of some frequencies. It can be concluded that the perforated plate could help to reduce the noise effectively.

Since inlet pressure is closely related to the flow status, it has great effects on SPL. The higher inlet pressure will lead to larger SPL of high frequency. And inlet pressure of 2.5 and 4.0 MPa have larger SPL at low frequency.

One limitation is that the studies are suitable for certain structure of HPRV and relationship between structural parameters and noises needs to be explored. Further analysis can be carried out to study flow characteristics and noise control performance of perforated plate, and to optimize its structure.
